# Relationships of physiologically equivalent temperature and hospital admissions due to I30–I51 other forms of heart disease in Germany in 2009–2011

**DOI:** 10.1007/s11356-015-5727-5

**Published:** 2015-12-01

**Authors:** Ivy Shiue, David R. Perkins, Nick Bearman

**Affiliations:** Faculty of Health and Life Sciences, Northumbria University, Newcastle upon Tyne, NE1 8ST England UK; Owens Institute of Behavioral Research, University of Georgia, Athens, GA USA; Center for Climate Change Communication, George Mason University, Fairfax, VA USA; School of Environmental Sciences, University of Liverpool, Liverpool, England UK

**Keywords:** Weather, Heart, Pericarditis, Cardiac arrest, Heart failure, Atrial fibrillation

## Abstract

We aimed to understand relationships of the weather as biometeorological and hospital admissions due to other forms of heart disease by subtypes, which have been paid less attention, in a national setting in recent years. This is an ecological study. Ten percent of daily hospital admissions of the included hospitals (*n* = 1618) across Germany that were available between 1 January 2009 and 31 December 2011 (*n* = 5,235,600) were extracted from Statistisches Bundesamt, Germany. We identified I30–I51 other forms of heart disease by the International Classification of Diseases version 10 as the study outcomes. Daily weather data from 64 weather stations that have covered 13 German states, including air temperature, humidity, wind speed, cloud cover, radiation flux and vapour pressure, were obtained and generated into physiologically equivalent temperature (PET). Admissions due to other diseases of pericardium, nonrheumatic mitral valve disorders, nonrheumatic aortic valve disorders, cardiomyopathy, atrioventricular and left bundle-branch block, other conduction disorders, atrial fibrillation and flutter, and other cardiac arrhythmias peaked when PET was between 0 and 10 °C. Complications and ill-defined descriptions of heart disease admissions peaked at PET 0 °C. Cardiac arrest and heart failure admissions peaked when PET was between 0 and −10 °C while the rest did not vary significantly. A common drop of admissions was found when PET was above 10 °C. More medical resources could have been needed for heart health on days when PETs were <10 °C than on other days. Adaptation to such weather change for medical professionals and the general public would seem to be imperative.

## Introduction

### Evidence before this study

Environmental factors have been central to many human chronic diseases, and the weather is no exception. The effect of the weather has been noted in scientific literature since the 1930s as increased hospital admissions due to coronary occlusion and heart failure were observed in correlation with low temperature that has prompted the concern on the influence of the seasonality effect (Bean and Mills 1938). The hypothesized mechanism was that an acute change in environmental temperature, being too cold or too hot (Bhaskaran et al. 2009), tends to increase myocardial oxygen consumption and may induce cardiac arrhythmias or an anginal attack (Ansari and Burch 1969; Epstein et al. 1969; Milo-Cotter et al. 2006). In addition, activation of the sympathetic nervous system and secretion of catecholamine could increase in response to low temperature that could be observed by the increased heart rate and peripheral vascular resistance (Hanna 1999). However, overall across the globe, conflicting results on the effect of the weather on human health outcomes have been presented in the literature.

### Knowledge gap

While there are complex interactions between the weather and human health outcomes that have been observed, methodological concerns on the risk assessment from previous research have been brought up recently (Modesti 2013). Seemingly, correlating air temperature and human health outcomes might not be adequate since there has been a difference between air temperature and the weather as biometeorological incorporating relevant meteorological parameters (Shiue and Matzarakis 2011). In addition, all the meteorological parameters are interacting with each other at the same time. Therefore, it would make no environmental or meteorological sense to only include air temperature or treat other climatic variables separately as the exposure. Since less attention has been paid to other forms of heart disease, compared to commonly known heart disease and cardiovascular disease, the present study would contribute to the scientific community with such evidence.

### Study aim

Following this context, we aimed to firstly investigate the monthly variations of hospital admissions due to other forms of heart disease and then to correlate with the weather as biometeorological in a national setting in recent years.

## Methods

### Study exposures and outcomes

This is an ecological study. Daily historical meteorological data including air temperature, humidity, wind speed, radiation flux, cloud cover and vapour pressure between 1 January 2009 and 31 December 2011 (three full calendar years) were obtained from the Federal Ministry of Transport, Building, and Urban Development (more details via http://www.dwd.de/). Daily hospital admissions with all diagnoses including emergency admissions in the same study period were extracted from the database held in Statistisches Bundesamt (more details via https://www.destatis.de/EN/Homepage.html), Wiesbaden, Germany. Statistisches Bundesamt randomly selects 10 % of hospital admissions (including first occurrence and recurrence) of each German hospital at the end of each year and stores the data for research purposes. Currently, there are 1618 hospitals in their record list. The admission (‘primary diagnosis’) is coded using the International Classification of Diseases version 10 codes (WHO 2009). In this study, we identified hospital admissions due to I30–I51 other forms of heart disease by subtypes (see Table 1) as the study outcomes, which were commonly seen in society, had enough number of events to be examined statistically and would protect any individual’s identity due to small numbers.

**Table 1:** Averaged numbers of monthly hospital admissions due to other forms of heart disease by subtypes in 2009–2011

	I30—Acute pericarditis	I31—Other diseases of pericardium	I33—Acute and subacute endocarditis	I34—Nonrheumatic mitral valve disorders	I35—Nonrheumatic aortic valve disorders	I38—Endocarditis, valve unspecified	I40—Acute myocarditis	I42—Cardiomyopathy
Jan	62 (9.0 %)	154 (8.1 %)	144 (8.5 %)	389 (9.6 %)	1366 (8.8 %)	31 (9.6 %)	94 (9.2 %)	659 (8.7 %)
Feb	50 (7.2 %)	145 (7.6 %)	151 (8.9 %)	320 (7.6 %)	1279 (8.3 %)	19 (5.9 %)	79 (7.8 %)	603 (8.0 %)
Mar	65 (9.4 %)	179 (9.4 %)	143 (8.4 %)	410 (10.1 %)	1468 (9.5 %)	29 (9.0 %)	110 (10.8 %)	721 (9.5 %)
Apr	61 (8.8 %)	163 (8.5 %)	140 (8.3 %)	360 (8.9 %)	1232 (9.0 %)	33 (10.2 %)	86 (8.5 %)	574 (7.6 %)
May	56 (8.1 %)	162 (8.5 %)	129 (7.6 %)	344 (8.5 %)	1369 (8.9 %)	23 (7.1 %)	83 (8.2 %)	664 (8.8 %)
June	61 (8.8 %)	152 (7.9 %)	140 (8.3 %)	302 (7.4 %)	1289 (8.3 %)	22 (6.8 %)	65 (6.4 %)	618 (8.2 %)
July	59 (8.5 %)	160 (8.4 %)	155 (9.1 %)	285 (7.0 %)	1296 (8.4 %)	27 (8.4 %)	78 (7.7 %)	612 (8.1 %)
Aug	66 (9.5 %)	137 (7.2 %)	156 (9.2 %)	312 (7.7 %)	1272 (8.2 %)	30 (9.3 %)	80 (7.9 %)	643 (8.5 %)
Sep	46 (6.6 %)	173 (9.0 %)	137 (8.1 %)	356 (8.8 %)	1256 (8.1 %)	28 (8.7 %)	76 (7.5 %)	664 (8.8 %)
Oct	50 (7.2 %)	157 (8.2 %)	147 (8.7 %)	364 (9.0 %)	1269 (8.0 %)	25 (7.7 %)	86 (8.5 %)	623 (8.2 %)
Nov	56 (8.1 %)	176 (9.2 %)	136 (8.0 %)	374 (9.2 %)	1362 (8.8 %)	25 (7.7 %)	85 (8.4 %)	673 (8.9 %)
Dec	61 (8.8 %)	156 (8.2 %)	119 (7.0 %)	253 (6.2 %)	1010 (6.5 %)	31 (9.6 %)	95 (9.3 %)	512 (6.8 %)
	I44—Atrioventricular and left bundle-branch block	I45—Other conduction disorders	I46—Cardiac arrest	I48—Atrial fibrillation and flutter	I49—Other cardiac arrhythmias	I50—Heart failure	I51—Complications and ill-defined descriptions of heart disease	
Jan	677 (8.2 %)	178 (8.4 %)	149 (9.4 %)	6113 (8.3 %)	1263 (8.4 %)	10,007 (9.1 %)	104 (7.1 %)	
Feb	659 (8.0 %)	174 (8.2 %)	126 (8.0 %)	6067 (8.2 %)	1197 (8.0 %)	9076 (8.2 %)	129 (8.8 %)	
Mar	747 (9.0 %)	220 (10.4 %)	114 (7.2 %)	6804 (9.2 %)	1413 (9.4 %)	10,418 (9.4 %)	154 (10.5 %)	
Apr	665 (8.1 %)	169 (8.0 %)	133 (8.4 %)	5942 (8.0 %)	1199 (8.0 %)	9522 (8.6 %)	121 (8.3 %)	
May	652 (7.9 %)	158 (7.5 %)	135 (8.5 %)	6035 (8.2 %)	1227 (8.2 %)	9494 (8.6 %)	121 (8.3 %)	
June	721 (8.7 %)	159 (7.5 %)	116 (7.3 %)	5937 (8.0 %)	1237 (8.2 %)	8982 (8.1 %)	101 (6.9 %)	
July	695 (8.4 %)	184 (8.7 %)	133 (8.4 %)	5968 (8.1 %)	1235 (8.2 %)	8399 (7.6 %)	128 (8.7 %)	
Aug	713 (8.6 %)	171 (8.1 %)	116 (7.3 %)	6119 (8.3 %)	1219 (8.1 %)	8491 (7.7 %)	121 (8.3 %)	
Sep	742 (9.0 %)	198 (9.4 %)	115 (7.3 %)	6185 (8.4 %)	1299 (8.7 %)	8491 (7.7 %)	110 (7.5 %)	
Oct	655 (7.9 %)	174 (8.2 %)	140 (8.8 %)	6285 (8.5 %)	1228 (8.8 %)	9091 (8.2 %)	129 (8.8 %)	
Nov	688 (8.3 %)	169 (8.0 %)	145 (9.2 %)	6718 (9.1 %)	1348 (9.0 %)	9492 (8.6 %)	141 (9.6 %)	
Dec	652 (7.9 %)	163 (7.7 %)	163 (10.3 %)	5925 (8.0 %)	1151 (7.7 %)	9088 (8.2 %)	108 (7.4 %)	

### PET calculation

**Fig. 1:**
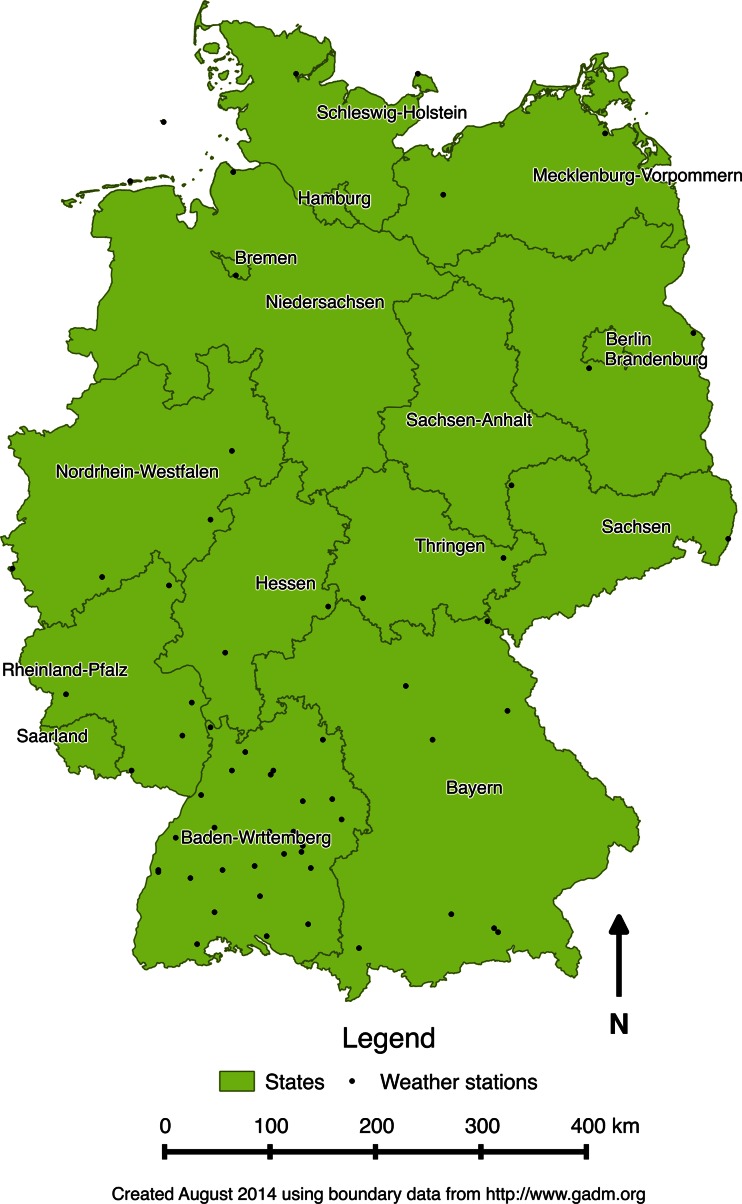
The included weather stations in Germany

In the first phase, to handle study exposures, we firstly used a geographic information system to map out the included weather stations (64 out of 78 representative stations across Germany based on the completeness of meteorological data) from each state of Germany. There are 16 states in total. For each of the included weather stations (see black dots shown in Fig. 1) in each German state, we generated all the included meteorological parameters mentioned above into a single index called physiologically equivalent temperature (PET) as the main study exposure to be correlated with hospital admissions. We then averaged daily PETs from each weather station for each German state. PET, with the widely known unit degrees Celsius, has been known to be used when considering the heat balance of the human body under standard conditions in an outdoor setting and initially created to characterize and evaluate the human bioclimate in a physiological setting (Höppe 1999). PET takes into consideration thermoregulatory processes such as sweat rate and blood vessel dilation and allows the user of the model to predict thermal attributes of the body such as sweat rate, core temperature and skin temperature. Calculating PET requires atmospheric, geographical and human-physiological inputs. In our case, we used the RayMan software for calculation (more details via http://www.mif.uni-freiburg.de/rayman/intro.htm). Atmospheric inputs include temperature, wind speed, humidity, sky cover and solar radiation. Geographical inputs include altitude and day length (assessed through latitude and longitude). Physiological inputs include gender, age, height, weight, amount of clothing and activity levels (measured in watts). Clothing values are seasonally adjusted for summer, winter and shoulder seasons, and all other physiological inputs are held constant based upon default model parameters. In the current analysis, 64 weather stations from 13 states with complete weather data were included for the statistical analysis. This has excluded three states containing Berlin, Saarland and Saxony-Anhalt without any of the meteorological parameters that would be needed for PET calculation.

### Statistical analysis

**Fig. 2:**
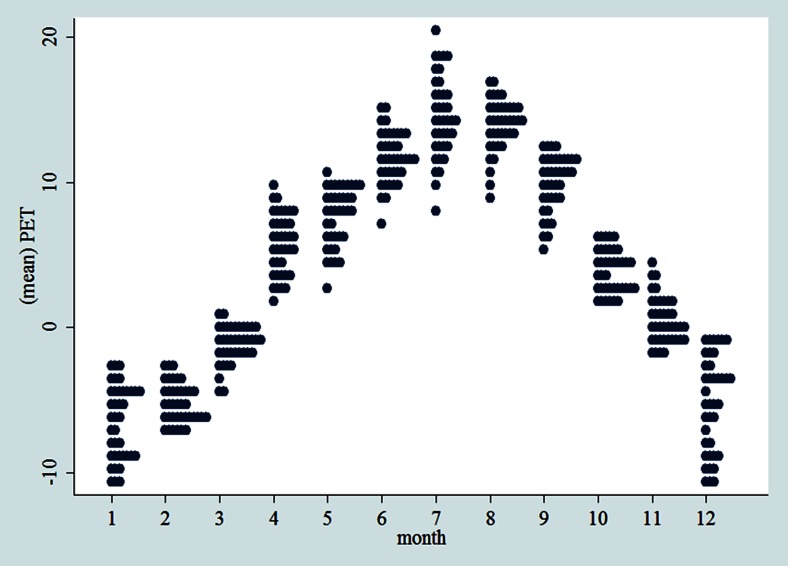
Averaged PET by month and by day over 3 years in 2009–2011

During the second phase, we plotted two-way fractional-polynomial prediction plots with confidence intervals (CI) in order to better describe the potential non-linear relationships between the weather and hospital admissions (the *y* axis in each figure denotes log-hazard ratios). Due to the German law prohibiting reporting of individual admissions and hospitals, correlational analysis was only made on a daily basis but not hourly. From the generated PETs in 2009–2011 (see Fig. 2), it was observed that there was no heat stress (PET >35 °C in Western Europe; Matzarakis and Mayer 1996). PETs were mainly between −10 and 20 °C throughout 3 years in 2009–2011. Statistical software STATA 12.0 version (STATA, College Station, TX, USA) was used to perform all the statistical analyses.

### Ethics approval and funding

This study was approved and supported by the EU FP-7 Data without Boundaries project (grant number 262608; more details via http://www.dwbproject.org/) and Statistisches Bundesamt, Germany. Since this study is only a secondary data analysis, no other ethics approval was required.

## Results

**Fig. 3:**
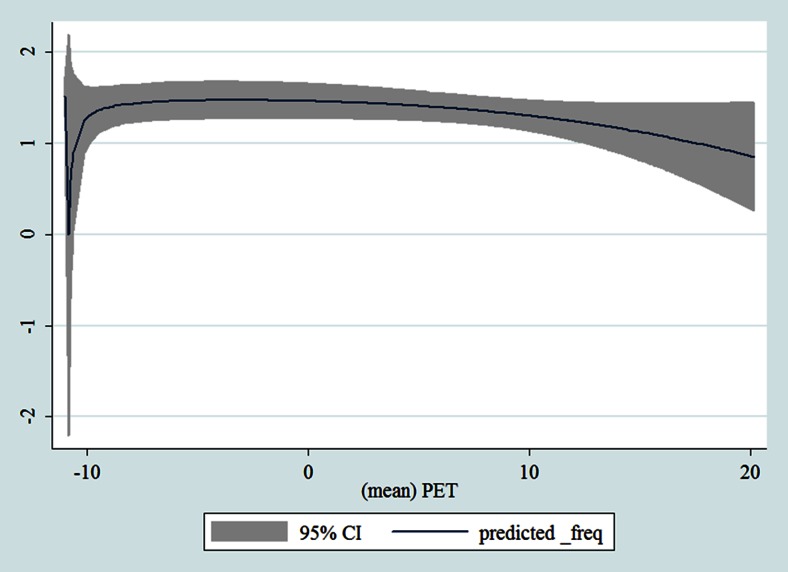
Relationship of PETs and admissions of I30—Acute pericarditis

**Fig. 4:**
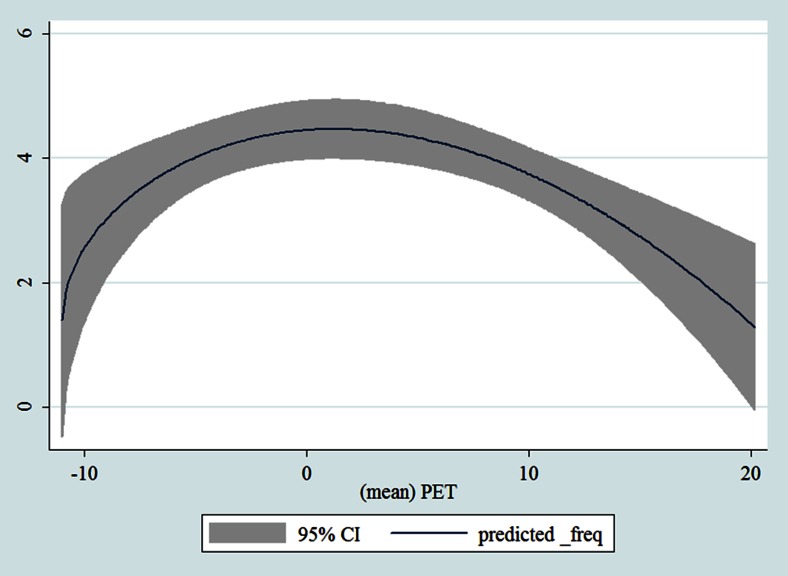
Relationship of PETs and admissions of I31—Other diseases of pericardium

**Fig. 5:**
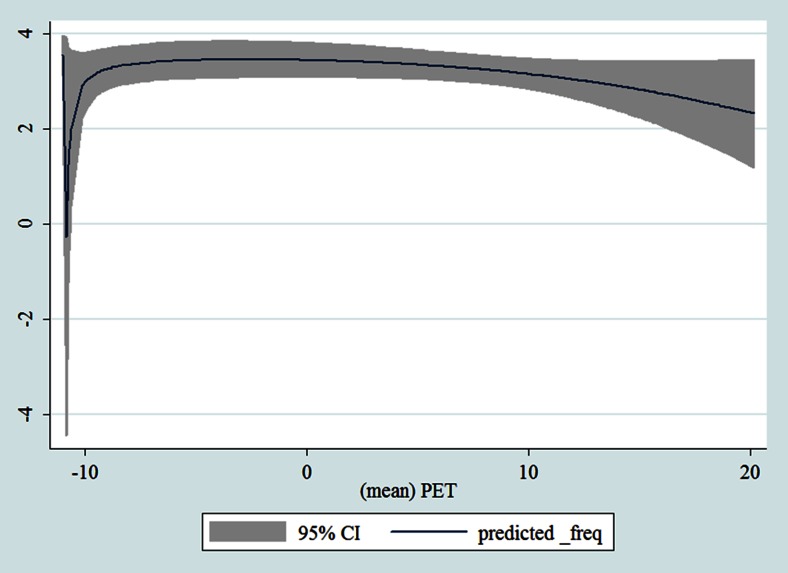
Relationship of PETs and admissions of I33—Acute and subacute endocarditis

**Fig. 6:**
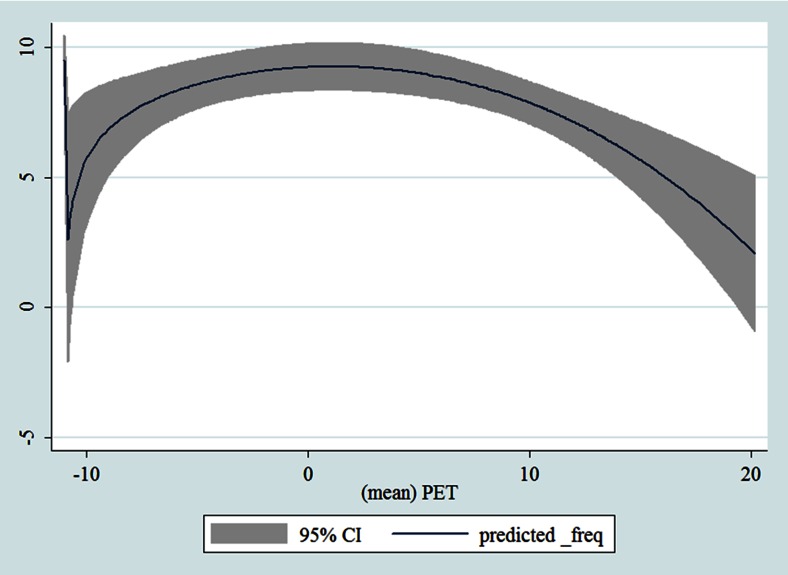
Relationship of PETs and admissions of I34—Nonrheumatic mitral valve disorders

**Fig. 7:**
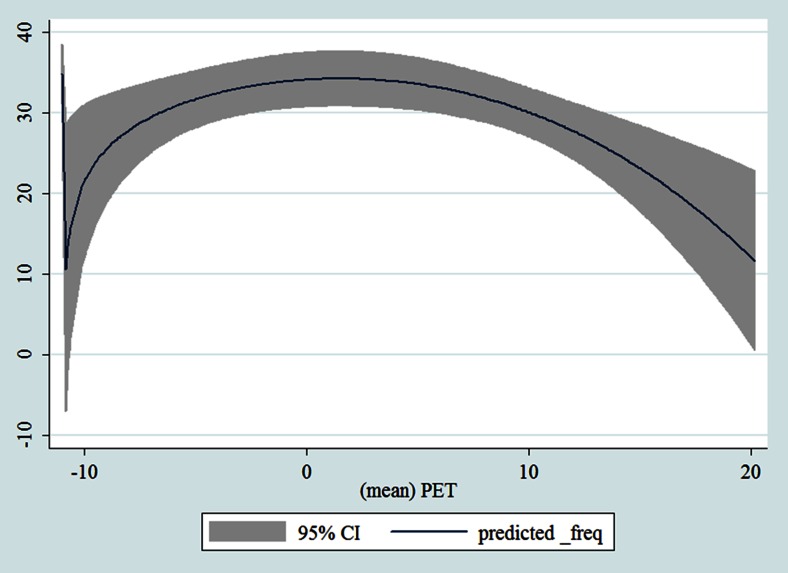
Relationship of PETs and admissions of I35—Nonrheumatic aortic valve disorders

**Fig. 8:**
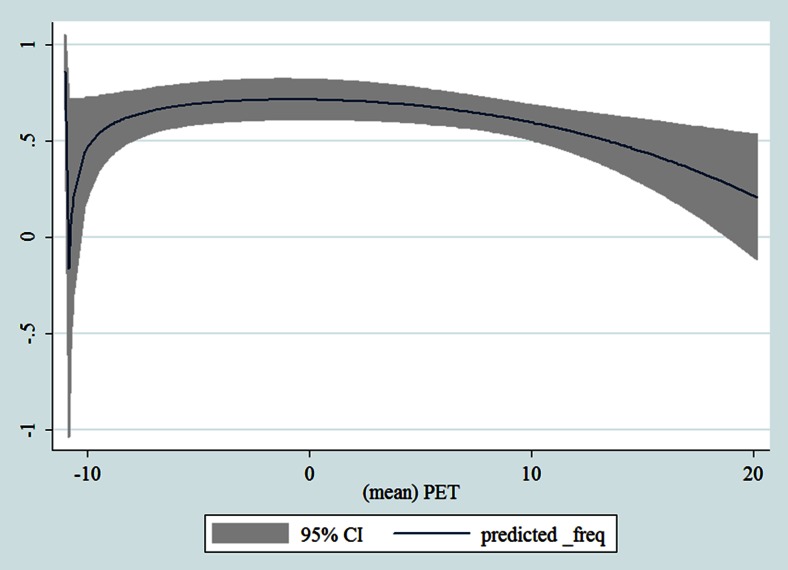
Relationship of PETs and admissions of I38—Endocarditis, valve unspecified

**Fig. 9:**
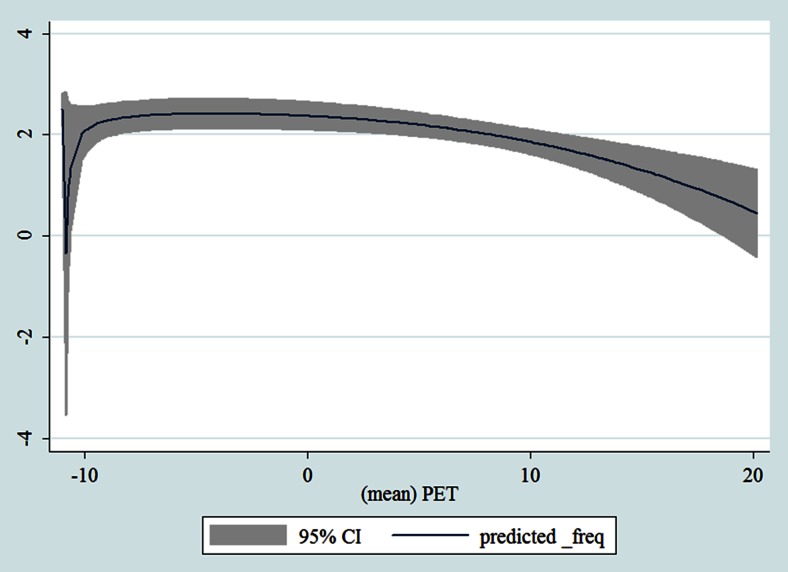
Relationship of PETs and admissions of I40—Acute myocarditis

**Fig. 10:**
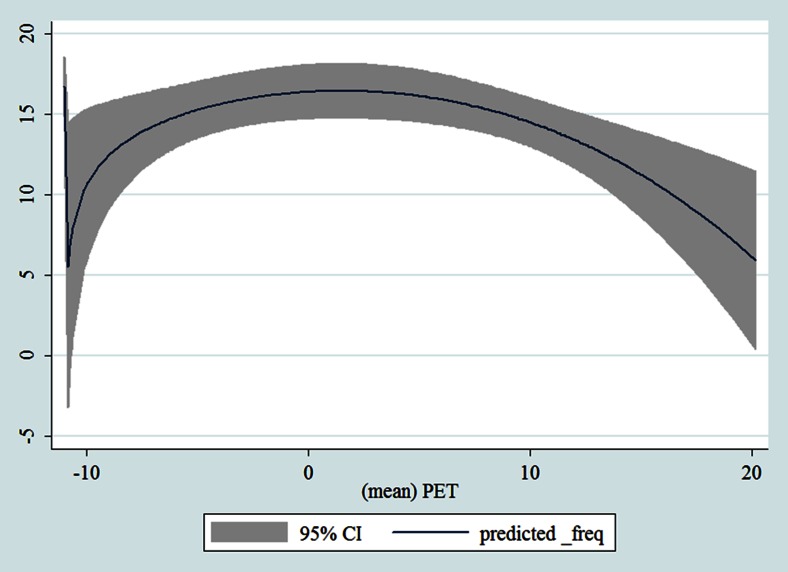
Relationship of PETs and admissions of I42—Cardiomyopathy

**Fig. 11:**
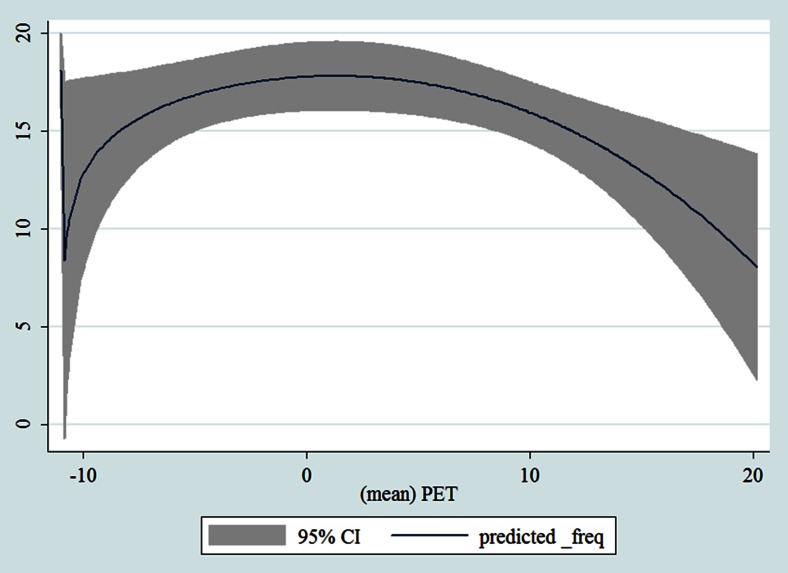
Relationship of PETs and admissions of I44—Atrioventricular and left bundle-branch block

**Fig. 12:**
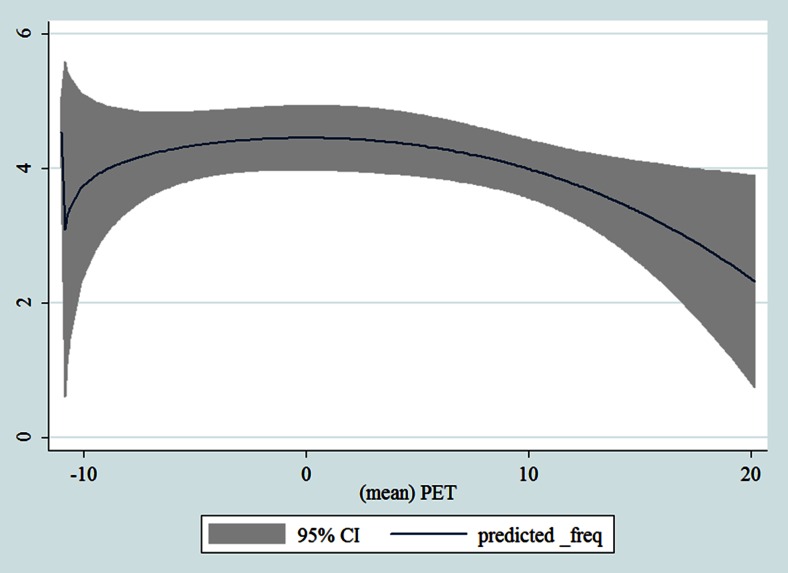
Relationship of PETs and admissions of I45—Other conduction disorders

**Fig. 13:**
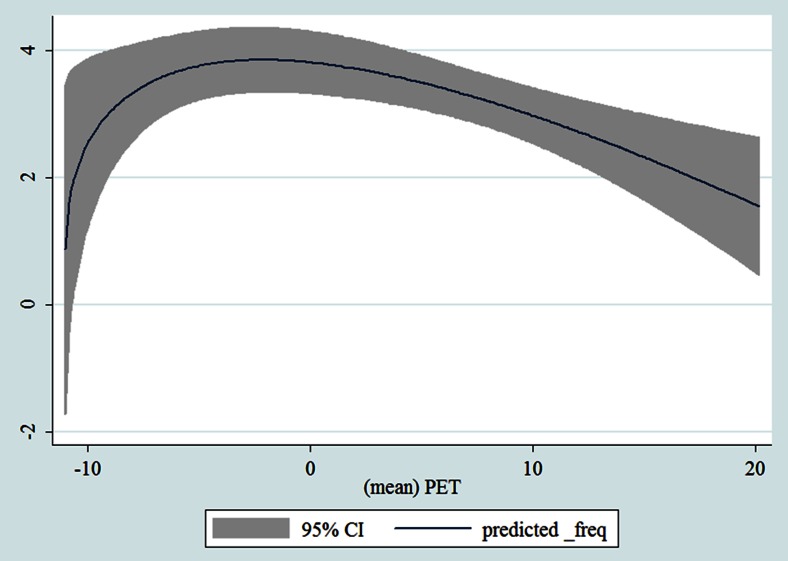
Relationship of PETs and admissions of I46—Cardiac arrest

**Fig. 14:**
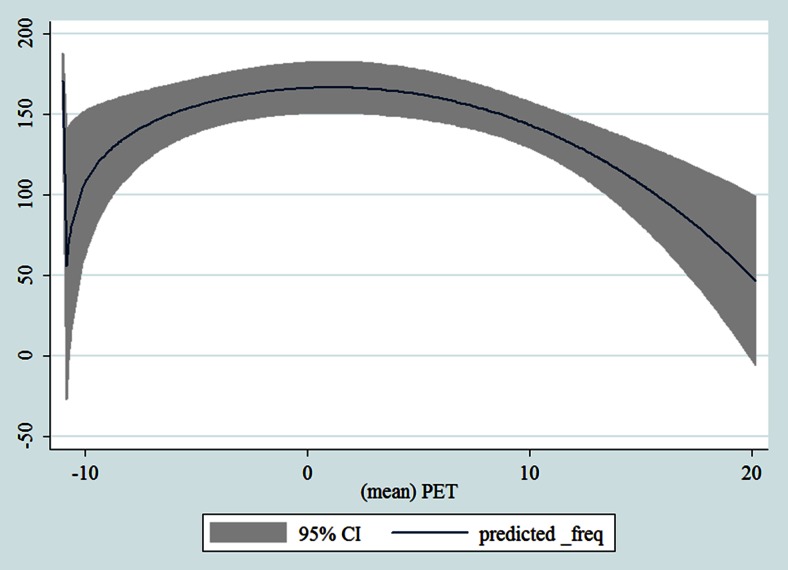
Relationships of PETs and hospital admissions due to I48—Atrial fibrillation and flutter

**Fig. 15:**
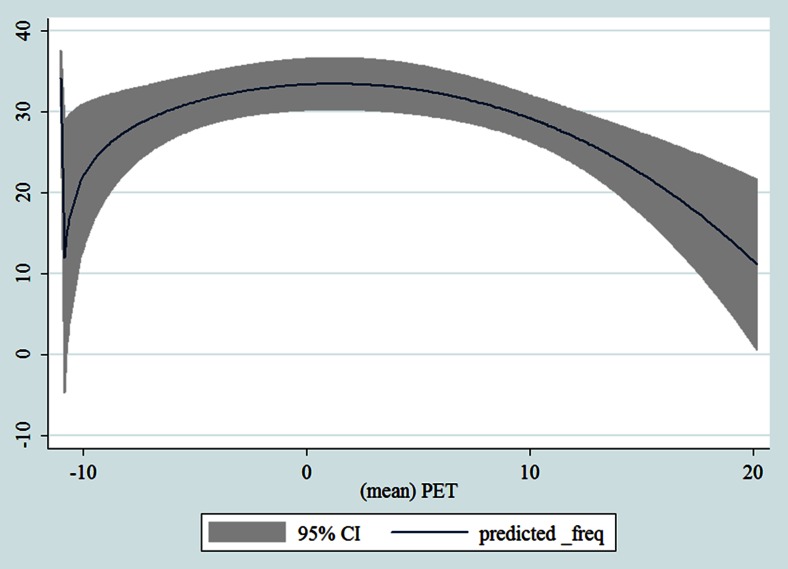
Relationships of PETs and hospital admissions due to I49—Other cardiac arrhythmias

**Fig. 16:**
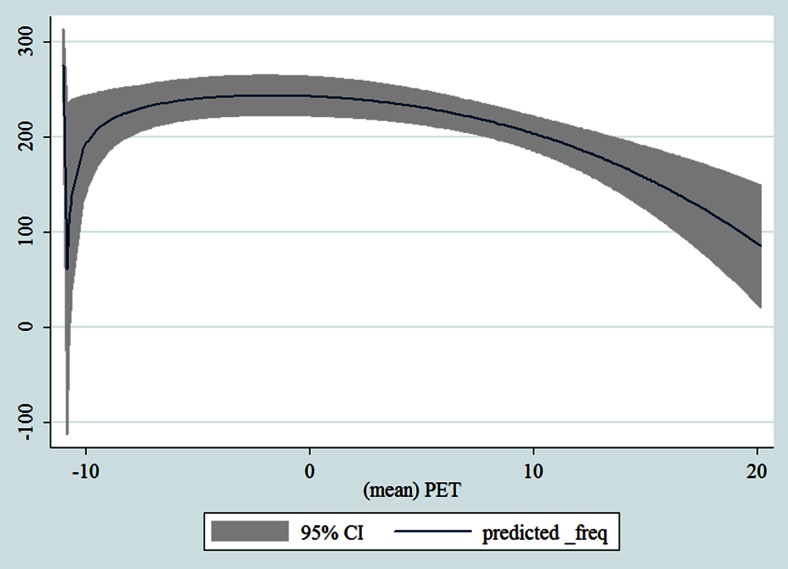
Relationships of PETs and hospital admissions due to I50—Heart failure

**Fig. 17:**
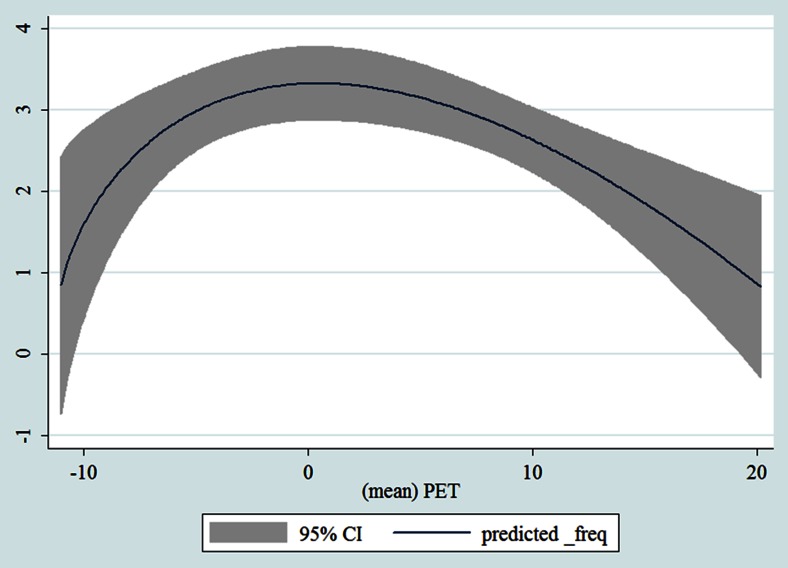
Relationships of PETs and hospital admissions due to I51—Complications and ill-defined descriptions of heart disease

Table 1 shows the monthly number of hospital admissions due to some common other forms of heart disease by subtypes. In general, for some subtypes, admissions peaked in winter and spring while the others did not seem to vary significantly. In Figs. 3, 4, 5, 6, 7, 8, 9, 10, 11, 12, 13, 14, 15, 16 and 17, the relationships of PETs and hospital admissions due to other forms of heart disease by subtypes are displayed accordingly. There could be four groups of phenomenon among these admissions. To be specific, admissions due to other diseases of pericardium, nonrheumatic mitral valve disorders, nonrheumatic aortic valve disorders, cardiomyopathy, atrioventricular and left bundle-branch block, other conduction disorders, atrial fibrillation and flutter, and other cardiac arrhythmias peaked when PET was between 0 and 10 °C. Complications and ill-defined descriptions of heart disease admissions peaked at PET 0 °C. Cardiac arrest and heart failure admissions peaked when PET was between 0 and −10 °C while the rest did not vary significantly. A common drop of admissions was found when PET was above 10 °C.

## Discussion

There has been only very few literature on the relationships between the weather and other forms of heart disease. In spite of relatively fewer admissions recorded in the population, the other reason might be the low variation of hospital admissions across seasons. Of note, we noted scientific literature on admissions, incidence or prevalence of human diseases but not death/mortality alone. This is because disease severity and treatment efficacy would be more important than the weather in relation to disease mortality as patients would have stayed in hospital during this period. Future research, either observational or experimental, looking into this topic should be encouraged.

### Weather and endocarditis

Back in the 1960s, animal research already observed the effect of exposure and acclimatization to cold on susceptibility of rats to bacterial endocarditis (Highman and Altland 1962). In a single hospital in Cleveland, USA, in 1993–2001 (Finkelhor et al. 2005), a study with a slightly larger human sample (*n* = 1279) found more admissions in the cold months as well. In a single hospital in Taipei, Taiwan, in 2003–2009 (Chen et al. 2012), cool seasons (temperature was averaged monthly) were observed to be associated with poor prognosis in human patients with infective endocarditis, although the sample size was rather small (*n* = 100). For some forms of heart disease such as myocarditis, fever that could occur more on the cold days or during the change of weather might explain some the peaks of hospital admissions.

### Weather and atrioventricular block

Literature on the relationship of the weather and atrioventricular block is much scarce. In a single hospital in Taipei, Taiwan, in 2004–2008 (Liu et al. 2011), a temperature (air temperature) change of greater than 11 °C within 30 days prior to admission was associated with a significantly higher proportion of patients with advanced atrioventricular block.

### Weather and cardiac arrest/arrhythmias

Although there seems to be more literature on the relationship of the weather and cardiac arrest, it was mostly focusing on out-of-hospital events, mortality or neurological conditions after cardiac arrest events. In Osaka, Japan, in 1998–2007 (Tanigawa-Sugihara et al. 2013), the number of out-of-hospital cardiac arrest events in 1 day was inversely correlated with the day’s mean atmospheric temperature, and the regression coefficient was greater on the days under 18 °C (*r* = −0.317, *P* < 0.001) than on days over 18 °C (*r* = −0.088, *P* < 0.001). In a single hospital in Vienna, Austria, in 1991–2010 (Stratil et al. 2013), nearly half of cardiac arrest patients were admitted to the hospital when the air temperature (meteorological data from four close weather stations) was <10 °C. In Sweden in 1990–1999 (Herlitz et al. 2002), admissions of cardiac arrest were observed to occur more in December and January when the cold months start. Literature on the relationship of the weather and cardiac arrhythmias is also very limited. In London, UK, in 1995–2003 (McGuinn et al. 2013), for every 1 °C decrease in ambient air temperature (meteorological data from all weather stations in southern England), risk of ventricular arrhythmias up to 7 days later increased by 1.2 % (95 % CI −0.6 %, 2.9 %). In 32 top-ranked hospitals in Beijing, China, in 2006–2010 (Xu et al. 2013), there was no seasonal variation of cardiac arrhythmia admissions seen in young patients but excess admissions in winter were seen in older adults (relative risk 1.67, 95 % CI 1.36–2.05).

### Weather and atrial fibrillation

Literature on the relationship of the weather and atrial fibrillation could be slightly more than those described in the previous paragraphs. For example, in 43 centres in the province of Buenos Aires, Argentina, in 2004–2006 (Fustinoni et al. 2013), hospital admissions due to atrial fibrillation were found to peak between air temperatures 5 and 9 °C. In a single Cardiac Care Unit located in Poland in 2005–2006 (Głuszak et al. 2008), the highest atrial fibrillation admissions were found in winter. Across Denmark, with half of the population in 1980–1993 (Frost et al. 2002), the risk of atrial fibrillation was modestly higher during the winter and was inversely associated with outdoor temperature. In a single hospital located in Toyoake, Japan, in 2001–2005 (Watanabe et al. 2007), admissions peaked in late autumn (relative risk 1.21, 95 % CI 1.16–1.27) as well. Similar results were obtained across the USA in 2000–2008 (Deshmukh et al. 2013). In Ontario province, Canada, in 1988–2001 (Upshur et al. 2004), the peaks of atrial fibrillation admissions were in April, and the troughs were in August. These previous observations were consistent with the finding from the present study.

### Weather and heart failure

Although literature on the relationship between the weather and heart failure seems to be slightly more than other subtypes of heart problems mentioned above as well, they were mostly with a focus on mortality. Here, we compared admission rates, incidence or prevalence only. In a single hospital in Tokyo, Japan, in 2004–2012 (Kaneko et al. 2014), admissions were observed to peak in winter and patients could be at a more vulnerable state. In a small teaching hospital in Urbana, USA, in 1997–2009 (Steinberg et al. 2012), heart failure admissions were found to occur more in September, October and November. Similar results were obtained in Ferrara Hospital located in Italy in 2002–2009 (Gallerani et al. 2011), and the authors also found that the effect was dependent on other known risk factors. In Niteroi, Brazil, in 1996–2004 (Jorge et al. 2009), the peak season for heart failure seemed to be late autumn. In the University of Uyo Hospital located in Southeastern Nigeria in 1998–2001 (Ansa et al. 2008), excess admissions due to heart failure were also recorded in the rainy season when the cold days came. In all hospitals within the Ninohe district, Japan, in 2002–2005 (Ogawa et al. 2007), however, more heart failure admissions were found to be in spring and winter.

### Strengths and limitations

The current study has a few strengths. First, our study is the first in assessing weather as biometeorological and hospital admissions due to other forms of heart disease by subtypes that have combined epidemiological, geographical and meteorological methods in Germany. Second, our data are limited to very recent years to ensure that we do not find significant statistical associations by chance alone through pooling decades of data. Third, we have drawn clear study catchments across Germany to ensure that both medical data and meteorological data can be matched geographically to rule out the potential ecological bias (i.e. one weather station plus numerous hospitals from other sub-regions). To be specific, in the current analysis, we ensured that in each German state, there could be at least one weather station to provide valid weather data to be correlated with hospital admissions within the state.

There were still other limitations worthy of being noted. First, we were unable to link with other population surveys to have adjusted covariates on lifestyle. In other words, this present study is at the ecological scale but not individual. Therefore, no causation could be drawn. By examining the correlations between the weather as biometeorological and hospital admissions, it is to indicate how much additional medical and social resources, such as medical professional time and hospital facilities (Shiue and Matzarakis 2011), might be anticipated with different days or seasons based on different PET values. Second, due to the restriction of the German law, we were only able to examine at the state level but not within smaller geographic regions. The lack of complete weather data in three German states has made our statistical analysis not perfectly complete at the national level. From the meteorological point of view, when investigating the weather effect, it would make scientific sense to generate climatic variables into a single index since they interact with each other at the same time and 1 °C in a cold climate would mean differently in a warm climate. Although there are other indexes such as Universal Thermal Climate Index (UTCI), the difference between UTCI and PET was not much (Shiue et al. 2014). In the current analysis, we did not include air pollution data due in part to the fact that by adjusting for air pollution in the epidemiological and statistical modelling, the effects could be stronger and easily reach statistical significance (Sabetghadam and Ahmadi-Givi 2014). The other practical reason was that the level of air pollution in Germany has been low in the recent years (more details via http://www.umweltbundesamt.de/en/data/current-concentrations-of-air-pollutants-in-germany). Therefore, the effect from air pollution would be minimal. There could also be indexes to be developed in the future that could additionally incorporate air pollutants since they could influence each other at the same time as well. However, this might be methodologically difficult since the level of each pollutant could vary across each geographic region. Clinically, in Germany, the waiting time between making appointments and actual admissions, if not emergency, could vary from days to months depending on specialization. However, such information was not available within the current dataset. Consequently, any lag effect of the weather was not available to be analysed in the present study. Future research with novel approaches to keep the strengths and to overcome the limitations would be highly suggested.

### Research, practice and policy implications

In sum, more hospital admissions due to other forms of heart disease by subtypes were observed on days with lower PET across Germany, although the causality cannot be confirmed due to the ecological study design in nature. However, nationally, how to prepare reallocation of medical and social resources in response to adapting to the change of weather, in particular at PET < 10 °C, would seem to be important. Previously, in the 2000s, one fourth of all Germans were found to believe that weather could influence their health to a “high degree” while one third were found to believe that weather could have “some influence on their health” (Höppe et al. 2002). In practice, the consideration of including the attitude from the general public and adaptation strategies in future public health programmes in order to save human capital in this country when facing climate change in the next decades would be suggested. For the future research direction, longitudinal monitoring on both the weather condition (with all meteorological parameters combined) and hospital admissions through observational or experimental study designs would be needed as well.
